# Interaction of dietary compounds, especially polyphenols, with the intestinal microbiota: a review

**DOI:** 10.1007/s00394-015-0852-y

**Published:** 2015-02-12

**Authors:** Aleksandra Duda-Chodak, Tomasz Tarko, Paweł Satora, Paweł Sroka

**Affiliations:** Department of Fermentation Technology and Technical Microbiology, Faculty of Food Technology, University of Agriculture in Krakow, ul. Balicka 122, 30-149 Kraków, Poland

**Keywords:** Bioactive metabolites, Bioavailability, Biotransformation, Digestion, Dysbiosis, Health impact

## Abstract

The intestinal microbiome plays an important role in the metabolism of chemical compounds found within food. Bacterial metabolites are different from those that can be generated by human enzymes because bacterial processes occur under anaerobic conditions and are based mainly on reactions of reduction and/or hydrolysis. In most cases, bacterial metabolism reduces the activity of dietary compounds; however, sometimes a specific product of bacterial transformation exhibits enhanced properties. Studies on the metabolism of polyphenols by the intestinal microbiota are crucial for understanding the role of these compounds and their impact on our health. This review article presents possible pathways of polyphenol metabolism by intestinal bacteria and describes the diet-derived bioactive metabolites produced by gut microbiota, with a particular emphasis on polyphenols and their potential impact on human health. Because the etiology of many diseases is largely correlated with the intestinal microbiome, a balance between the host immune system and the commensal gut microbiota is crucial for maintaining health. Diet-related and age-related changes in the human intestinal microbiome and their consequences are summarized in the paper.

## Introduction

For many years, it was thought that the function of the large intestine was to reabsorb water and salt and remove unused food debris. It is now evident that the intestinal microbiota plays an important, if not crucial, role in the metabolism of chemical compounds found within foods. The large number of bacteria inhabiting the large intestine forms a highly complex ecosystem called the ‘intestinal microbiome’. The word ‘microbiome’ was first introduced in 2001 to define the collective genomes of the microbiota [1]. In 2007, the ‘human microbiome project’ was launched with the aim of collecting and integrating genomic information from many diverse human microbiomes and examining the relationship between changes in the human microbiome and various diseases [2, 3]. Growing scientific interest in the composition of the microbiome and its impact on human health has led to the initiation of many similar projects all over the world. The NIH Human Microbiome Project (2007, USA) aimed to characterize the influence of the microbiota on the human body and to correlate changes in these microbial populations with human health. The Irish ELDERMET Project (2007–2013) focused on characterization of the fecal microbiota associated with aging and aimed to correlate the composition, diversity and metabolic potential of the fecal microbial metagenome with health, diet and lifestyle. MetaHIT (metagenomics of the human intestinal tract, 2008–2011) was a European project focused on assessing the role of the microbiota in inflammatory bowel disease and obesity. The French project Microbes (human intestinal microbiome in obesity and nutritional transition, 2008–2010) sought to identify metagenomic signatures that characterize the relationship between the intestinal microbiome and the nutritional and metabolic status of the host. The interactions between intestinal microbes and host health were also evaluated by the Australian jumpstart human microbiome project (2009) and Canadian Human Microbiome Initiative (2009). Still in progress is the Korean Microbiome Diversity using Korean Twin Cohort Project (2010–2015) that aims to determine the microbiomes on various epithelial sites of the human body using the Korean twin cohort and to investigate the relationship between the human microbiome and disease.

The aim of this article is to review recent studies on factors affecting the composition of the human gut microbiota with a particular emphasis on polyphenolic compounds. The manuscript presents a compilation of the knowledge on functional aspects of the normal gut microbiota from two perspectives: (1) the role of particular microbial species in the metabolism of polyphenols and (2) a detailed insight into the role of biotransformation of polyphenols in relation to their beneficial functions on health.

## Microbial diversity of the human gut microbiota

The human gut is colonized by an enormous number of microorganisms, mainly bacteria. It is estimated that the microbiota of a human adult is composed of ~10^14^ bacterial cells, which is ten times more than the total number of human cells [4]. The metabolic capacity of the intestinal microbiome is approximately 100-fold greater than that of the human liver. This is a result of the great diversity of bacterial species forming the population, and hence the large number of genes which they contain [4].

The process of gut colonization starts immediately after birth. The infant’s microbiota initially shows instability and low diversity, evolving into a more stable adult-type microbiota after the first 2–4 years of life. Its final composition depends on environmental factors. According to Sakata et al. [5] in the first week of human life enterobacteria, streptococci, enterococci and staphylococci are already present in the infant’s gut while anaerobes such as bifidobacteria, lactobacilli and *Bacteroides* spp. are not. The gradual consumption of oxygen in the intestine by aerobic microorganisms provides conditions for the settlement of anaerobic bacteria. Breastfeeding is a significant factor in the determination of the neonatal gut microbiota. In full-time breastfed babies (1 month old), bifidobacteria constituted a predominant group in the feces, while in formula-fed infants, although bifidobacteria were also the predominant species, other bacteria such as enterobacteria, enterococci, lactobacilli, clostridia and *Bacteroides* spp. were present in significantly higher numbers compared with breastfed infants [6, 7].

Initial colonization of the intestine does not appear to be random but rather ‘pre-programmed’. Although host genetics can predict microbial composition to an extent, several extrinsic factors (mode of infant delivery, antibiotic exposure, neonatal nutrition, adult nutrition, stress, age, degree of hygiene, bacterial infections) contribute to the development of an individual’s unique microbial composition and as a result, susceptibility to several diseases [8, 9]. It has been shown that infants born vaginally acquire their own mother’s vaginal and intestinal flora (dominated by *Lactobacillus*, *Prevotella*, *Atopobium* or *Sneathia* spp.) and have higher levels of *Clostridium* spp, while infants delivered by cesarean section have increased levels of skin-associated bacteria including *Staphylococcus*, *Corynebacterium* and *Propionibacterium* spp. [7, 10, 11].

The microbiota of the human gastrointestinal tract starts in the mouth, and the number of viable cells is estimated at 10^8^–10^10^ colony-forming units (CFU) of bacteria per gram of saliva. As a result of the swallowing reflex, these bacteria are continuously transferred to the subsequent parts of the gastrointestinal tract. The number of microorganisms is significantly reduced in the stomach (~10^3^ CFU/g gastric juice), duodenum and jejunum (10^2^–10^4^ CFU/g content), increasing again in the ileum and colon (approximately 10^10^ CFU/g content and 10^10^–10^12^ CFU/g content, respectively) [12]. The enormous number of microorganisms that inhabit the human large intestine (up to 10^12^ bacteria per gram of colonic content) is the highest accumulation of microorganisms in the environment that has been reported to date [13].

The composition, as well as the ratio of different species that form the intestinal microbiome, is very diverse within the human population [14]. The composition of the human gut ecosystem is influenced by multiple and diverse factors, such as age, origin, environment, dietary habits (including probiotics) and the application of antibiotics. Hence, each individual has his or her own unique profile of microbial species, which can be compared to a fingerprint. Owing to the multitude of direct and indirect interactions with the host organism, the intestinal microbiome is closely linked to the health of the host [15, 16].

Evidence from culture-based studies led to the number of bacterial species in the human intestinal microbiome being estimated at approximately 400–500. However, only a small proportion of bacteria can be easily cultivated in vitro. Consequently, the major populations identified in stool samples were composed of bacteria that can grow quickly in classical high-nutrient growth media at mesophilic temperatures. Moreover, such studies were usually conducted in anaerobic conditions, whereas some gut bacteria prefer microaerophilic growth. Development of molecular methods of bacterial diversity assessment has enabled these limitations to be overcome. Since the year 2000, large-scale 16S rRNA or metagenomic studies have allowed scientists to improve their knowledge regarding the diversity of the human gut microbiome [17, 18]. It is commonly accepted that ~80 % of the bacteria identified by molecular tools in the human gut are uncultured and hence can be characterized only by metagenomic studies [17, 19]. Qin et al. [20] found that there are 1,000–1,150 prevalent bacterial species, and each individual harbors at least 160 species.

There are significant inter-individual differences in the bacterial species found in the gastrointestinal tract, and these differences are a result of the age, health, diet or geographical location of the individual. On the basis of 16S rRNA analysis of intestinal microbiota samples taken from different individuals, it has been shown that, despite the great diversity of bacterial species, the majority (98 % of all species) belong to only four bacterial phyla: *Firmicutes* (64 %), *Bacteroidetes* (23 %), *Proteobacteria* (8 %) and *Actinobacteria* (3 %), whereas other minor taxonomic divisions are quite diverse [4, 21, 22]. It should be emphasized that the ratio between these groups is strongly dependent on the location within the intestine, and on the ethnicity of the host. *Firmicutes* and *Bacteroidetes* dominate in the large intestine, while in the jejunum *Proteobacteria* are more abundant than *Bacteroidetes*. In the ileum of Japanese adults, no *Bacteroidetes* were detected, whereas these bacteria dominated the microbiota of healthy Swedish women [12, 23, 24].

## Changes of the intestinal microbiota

### Age-related changes

The microbiota composition is not stable over the life of an individual. It is natural that the diet of elderly individuals changes for many reasons, including loss of sensation of taste and smell, tooth loss, and chewing difficulties, or due to certain diseases that result in the exclusion of certain components from the diet (cholesterol, sugar, salt). Some of these age-related microbiota composition changes may therefore be attributable to diet. However, composition of the intestinal microbiota may also vary in elderly subjects independently of diet because of a number of physiological and immunological factors, such as reduced functionality of the immune system. It has been shown that the microbiota associated with elderly individuals is characterized by reduced abundance of *Ruminococcus* and *Blautia* spp. and a diminished abundance of several butyrate producers, while the number of *Escherichia* is increased compared with young controls [25]. The ratio of *Firmicutes* over *Bacteroidetes* (F/B) is one of the parameters known to change throughout the lifespan. The ratio is lower in the first year of life (0.4), increases in adulthood (10.9), and decreases during old age (0.6) [26]. Recently, it has been demonstrated that the gut microbiota in adolescent children 11–18 years of age differs from that of adults, with significantly higher abundance of *Bifidobacterium* and *Clostridium* genera [27]. These results indicate that adolescent children should be considered a separate age-group in this field of research.

It is notable that the ratio of *Firmicutes* to *Bacteroidetes* is higher in obese than in lean humans. Moreover, this proportion decreases with weight loss on a low-calorie diet [28]. This means that in addition to the effect of excess calories per se, a high-calorie diet predisposes to obesity through microbiota modulation, which manifests by the increased F/B ratio. Hence, the F/B ratio may also be considered a useful biomarker of obesity [29].

### Diet-related dysbiosis

A balance between the host immune system and the commensal gut microbiota is crucial for maintaining health. If this balance is disturbed (dysbiosis), the host–microbe relationship can progress toward disease [14, 30]. Dysbiosis can be diet-related [19]. It is now clear that microbes present in the human gut are essential for the process of digestion in the host. During anaerobic fermentation, their metabolism causes a breakdown of indigestible compounds such as resistant starch and plant polysaccharides, which results in short-chain fatty acid (SCFA) production. Other examples of microbe-specific metabolism are the syntheses of vitamins and amino acids [14]. In contrast, some compounds present in the diet can modulate the microbiota composition, resulting in changes in the metabolic activity of intestinal bacteria [31].

### Specific diets

The composition of the gut microbiota is susceptible to the quality and quantity of ingested carbohydrates that are the main carbon and energy source for the microbes. Any diet that is either selective or defective with respect to its nutrient content will cause the disruption of the delicate balance between the host and its intestinal microbiota, leading to diet-related dysbiosis. Such a situation may subsequently favor the overgrowth of opportunistic pathogens and weaken the host defense against infection and chronic inflammation, possibly via alterations in mucosal immunity.

In studies of the intestinal microbiota in humans with celiac disease, the numbers of aerobic *Staphylococcus*, as well as anaerobic *Clostridium* and *Bacteroides*, were significantly higher (*p* < 0.05) than in healthy subjects. In contrast, the number of *Bifidobacteria* was slightly higher in healthy controls [32]. A gluten-free diet (GFD) is a type of diet used for celiac disease treatment. It has been shown that a GFD itself could lead to modifications of the composition and immune properties of the gut microbiota. In the study of Sanz [33], ten healthy subjects (30.3 years old) consumed a GFD for 1 month by replacing gluten-containing foods that they usually ate with certified gluten-free foods. Analyses of their fecal microbiota and dietary intakes demonstrated that populations of *Bifidobacterium* and *Lactobacillus* (generally regarded as healthy bacteria) decreased, while populations of potentially unfavorable bacteria such as *Escherichia coli* and total *Enterobacteriaceae* increased; this occurred in parallel with reductions in the intake of polysaccharides. A diet rich in the fructan-type resistant starches, especially oligofructose and inulin, is known to promote “good” species of colon bacteria [34]. Such prebiotics are found in the diet mainly in association with wheat, barley and onions [35]. This means that a GFD could cause similar, potentially adverse changes in the microbiota solely on the basis of a significant reduction in fructan intake. Provision of gluten-free but prebiotic-rich foods, or diet supplementation with fructan-type prebiotics, could help avoid these adverse effects and provide important support to the intestinal microbiota of people with celiac disease.

Other researchers have focused on differences in the fecal microbiota of neonates that were fed either breast milk or formula. Many studies have shown that breastfeeding results in the development of an intestinal microflora rich in *Bifidobacteria* and *Lactobacillus*, while formula-fed infants are colonized more often with *E. coli*, *Clostridium difficile*, members of the *Bacteroides fragilis* group and *Lactobacilli* [6, 7, 36].

The impact of diet on microbiota composition was also examined by De Filippo et al. [29]. They demonstrated that the fecal microbiota in a cohort of Italian children was different to that found in children in a rural village in Burkina Faso (BF). BF children showed a significant enrichment in *Bacteroidetes* and depletion in *Firmicutes*, with a unique abundance of bacteria from the genera *Prevotella*, *Treponema* and *Xylanibacter*, known to contain a set of bacterial genes for cellulose and xylan hydrolysis, which were completely lacking in the Italian children. In addition, significantly more SCFAs were found in BF than in Italian children, whereas *Enterobacteriaceae* (*Shigella* and *Escherichia*) were significantly underrepresented. The authors hypothesized that the gut microbiota have coevolved with the long-term polysaccharide-rich diet of BF individuals, allowing them to maximize energy intake from dietary fiber, and protecting against inflammation and noninfectious colonic disease. They also suggested a potential impact of the “Western diet” on the colonization and establishment of the intestinal microbiota in European and other developed countries.

Similar adaptation of microbiota to a specific diet was demonstrated in the gut microbiome of Japanese individuals, which is the only microbiome to date shown to possess the porphyranase genes. Hehemann et al. [37] suggested that those enzymes, which are involved in the carbohydrate metabolic pathway of porphyran from marine red algae, were most likely acquired by the human gut microbiota through horizontal gene transfer from marine microbes. These results are concordant with the fact that Japanese people have a tradition of eating foods containing raw seaweed (such as nori, the edible seaweed of the red algae genus *Porphyra* that is used in preparing sushi) and this is suggested as the reason for the presence of porphyranases in Japanese but not American gut microbiota [37, 38].

Another example of when long-term dietary practices affect the composition of the resident microbiota was demonstrated for developed countries and the so-called Western diet, which is high in sugar and fat. This diet can cause dysbiosis by increasing the number of *Clostridium innocuum*, *Eubacterium dolichum*, *Catenibacterium mitsuokai* and *Enterococcus* spp. and decreasing *Bifidobacteria* and *Bacteroidetes* [9, 39, 40]. Wu et al. [41] showed that the *Bacteroides* enterotype was highly associated with a diet rich in animal protein, particular types of amino acids and saturated fats, which suggests that meat consumption (as in a Western diet) characterized this enterotype. In contrast, the *Prevotella* enterotype was associated with high values for carbohydrates and simple sugars, indicating association with a carbohydrate-based diet more typical of agrarian societies. These results confirmed conclusions from a previous study, in which overweight adolescents were subjected to a calorie-restricted diet (10–40 %) and increased physical activity (15–23 kcal/kg body weight/wk) over 10 weeks [42]. The intervention led to increased numbers of *B. fragilis* and *Lactobacillus* groups, and decreased numbers of the *Clostridium coccoides* group, *Bifidobacterium longum* and *B. adolescentis*. The role of changes in gut composition in the development of obesity was examined in an Egyptian population [43]. The results confirmed that the numbers of bacteria from the phyla *Firmicutes* and *Bacteroidetes* were statistically significantly increased in the obese group compared with the normal weight group. Moreover, a trend for subjects with high fat intake to have positive *Firmicutes*, and those with the highest carbohydrate intake to have positive *Bacteroidetes* and *Firmicutes*, was also observed.

Vegetarianism is a diet that can modulate the intestinal microbiota in humans because of the high amounts of fiber consumed. It results in increased SCFA production by microbes [44], which can decrease intestinal pH. Indeed, subjects on a vegan or vegetarian diet showed significantly lower intestinal and stool pH, which prevented the growth of potentially pathogenic bacteria such as *E. coli* and other members of the *Enterobacteriaceae* [45]. Significantly lower total counts of *Bacteroides* spp., *Bifidobacterium* spp., *E.* *coli* and *Enterobacteriaceae* spp. in vegan samples was demonstrated compared with controls. Enrichment of *Prevotella* versus *Bacteroides* was also shown for vegetarians as well as for individuals who consume a high proportion of fruit and vegetables and a low proportion of meat. The reverse was associated with a diet that contains a low proportion of plant-based foods [25, 41]. Dietary interventions designed to promote SCFA production in the colon may therefore have some efficacy in the treatment of dysbiosis.

## Impact of diet-derived nutraceuticals on the microbiota

### Polyphenols

Many studies have examined the potential effects of polyphenols against pathogens. However, there are notably few studies investigating the influence of polyphenols on the composition and activity of the nonpathogenic gut microbial community. The most potent inhibitors of microorganism growth are probably the polyphenolic compounds from green and black tea. It has been shown that the bioactive components of tea, which include epigallocatechin gallate, epicatechin gallate, epigallocatechin, gallocatechin, epicatechin and catechin [46], can inhibit the growth of many pathogens including *Helicobacter pylori* [47], *Staphylococcus aureus*, *E. coli* O157:H7 [48, 49], *Salmonella typhimurium* DT104, *Listeria monocytogenes*, methicillin-resistant *S. aureus* [50, 51], *Pseudomonas aeruginosa* [52], hepatitis C virus [53], influenza virus [54], HIV [55–57], Epstein–Barr virus [53] and fungi of the *Candida* genus [58]. Some studies suggest that polyphenols can stimulate the growth of commensal and beneficial microbiota while pathogenic strains are inhibited. For example, the growth of certain pathogenic bacteria such as *Clostridium perfringens*, *C. difficile* and *Bacteroides* spp. was significantly inhibited by tea phenolics and their derivatives, while commensal anaerobes such as *Clostridium* spp., *Bifidobacterium* spp. and probiotics such as *Lactobacillus* spp. were less severely affected [59].

The inhibitory effect of citrus polyphenols such as hesperetin, naringenin, poncirin and diosmetin on the growth of *H. pylori* [60] has also been demonstrated. Parkar et al. [61] investigated the effect of common dietary polyphenols on the growth of the human gut bacteria and their adhesion to enterocytes. All of the polyphenols analyzed, except rutin, were found to affect the viability of representative gut microflora in vitro at doses likely to be present in the gastrointestinal tract, but to different degrees. Naringenin and quercetin were the most active with the lowest minimum inhibitory concentrations for all of the four bacteria tested *(Lactobacillus rhamnosus*, *E. coli*, *S. aureus* and *S. typhimurium*). Naringenin and phloridzin were the most effective inhibitors of *S. typhimurium* adherence to Caco-2 enterocytes, while phloridzin and rutin enhanced the adherence of the probiotic *L. rhamnosus*. Polyphenols thus appear to be able to alter gut microecology and, by affecting the total number of beneficial species in the gut, may confer positive gut health benefits. In another study, it was demonstrated that both rutin and quercetin inhibited the growth of the pathogenic bacteria *E. coli* and *Serratia marcescens*, while only quercetin had an inhibitory impact on *Klebsiella pneumonia* and *Proteus vulgaris* [62]. Differences between the actions of polyphenols belonging to the same group (flavonols or flavanones) are probably dependent on the 4-carbonyl group in the C ring of the flavonoid skeleton. The results of Duda-Chodak [31] suggest that the presence of this group is critical for the inhibitory activity of flavonols and flavanone aglycones. It was demonstrated that flavonoid aglycones (at doses 4–250 μg/ml), but not their glycosides, may inhibit growth of some intestinal bacteria. In this study, rutin had no inhibitory influence on the intestinal bacteria analyzed, and even slight stimulation of the growth of *Lactobacillus* spp. was observed. In contrast, its aglycone quercetin exerted a dose-depended inhibitory effect (except on *Bifidobacterium catenulatum)*, and this was especially strong on *Ruminococcus gauvreauii*, *Bacteroides galacturonicus* and *Lactobacillus* spp. (MIC 20–50 μg/ml) growth. The same was true for flavanones. Naringin and hesperidin (flavanone glycosides) had no impact, but their aglycones (naringenin and hesperetin, respectively) inhibited growth of almost all bacteria analyzed (MIC ≥ 250 μg/ml). In the same study, catechin had no influence on tested representatives of human intestinal microbiota. (+)-Catechin has been shown to significantly inhibit growth of *Clostridium histolyticum* [63]; however, the impact of catechin on *Clostridium* depends on the species. In cultures with (+)-catechin, statistically significant increases in the growth of the *C. coccoides*–*Eubacterium rectale* group, *Bifidobacterium* spp. and *E. coli*, as well as a significant inhibitory effect on the growth of the *C. histolyticum* group, have been shown [63]. The significant changes in bacterial composition in response to (+)-catechin were accompanied by its rapid conversion to (+)-epicatechin. In contrast, the effect of (–)-epicatechin was less profound, only significantly increasing the growth of the *C. coccoides*–*E. rectale* group.

The growth of *Clostridium* spp. can be decreased significantly by a tannin-rich diet or red wine polyphenols, while *Bacteroides* and *Lactobacillus* will be stimulated in such conditions [64, 65]. In the study of Sanchez-Patan et al. [66], an extract of wine phenolics (rich in quercetin, flavan-3-ols and anthocyanins) had no influence on the *Lactobacillus/Enterococcus* spp., *Bacteroides* spp., *Bifidobacterium* spp. and members of the domain bacteria. An observed slight inhibition in the *C. histolyticum* group was not significant. However, red wine polyphenols can influence the gut microbiota. The daily consumption of red wine polyphenols for 4 weeks significantly increased the number of *Enterococcus*, *Prevotella*, *Bacteroides*, *Bifidobacterium*, *Bacteroides uniformis*, *Eggerthella lenta* and *Blautia coccoides*–*E. rectale* groups [67].

Flavonols can also modulate the gut microbiota by affecting the adhesion of bacteria to intestinal cells, but the influence of flavan-3-ols on bacterial adhesion differs greatly between compounds, strains and degree of differentiation of intestinal cells [68]. All flavan-3-ols tested significantly inhibited adhesion of *Lactobacillus acidophilus* LA-5 and *Lactobacillus plantarum* IFPL379, except epigallocatechin gallate, which enhanced *L. acidophilus* LA-5 adhesion to Caco-2 cells. Procyanidins B1 and B2 markedly increased the adhesion of *Lactobacillus casei* LC115 to HT-29 cells, whereas epigallocatechin increased *L. casei* LC115 adhesion to Caco-2 cells.

There are only a few studies that have examined the antibacterial properties of anthocyanins. The anthocyanidins pelargonidin, delphinidin and cyanidin, as well as cyanidin-3-glucoside (C3G), have been shown to inhibit growth of *E. coli* CM 871, but had no effect on strains of the *Lactobacillus* and *Bifidobacterium* genera, *Salmonella enterica* SH-5014 or *Enterococcus faecalis* E-203 [69]. It was also found that C3G inhibited secretion of CagA and VacA in *H.* *pylori* via down-regulation of secA expression [70]. This means that a diet abundant in various berries rich in C3G may be beneficial in reducing gastric inflammation or stomach cancer if these are dependent on *H. pylori* infection. In another study, all of the anthocyanins tested significantly enhanced the growth of *Bifidobacterium* spp. and *Lactobacillus*–*Enterococcus* spp., suggesting that anthocyanins and their metabolites may exert a positive effect on the intestinal bacterial population [71].

Anthocyanins have been shown to stimulate the growth of lactic acid bacteria and increase malolactic fermentation [72]. These bacteria can cleave the anthocyanin molecules and use the sugar moiety as a carbohydrate source. Cranberries, a fruit rich in proanthocyanidins, are used for maintaining urinary tract health. It is thought that the beneficial effect of cranberry is due to antiadhesion activity against both antibiotic-susceptible and antibiotic-resistant strains of uropathogenic P-fimbriated *E. coli* [73]. It has been shown that cranberry proanthocyanidins have unusual A-type linkages [74] compared with the more common B-type linkages found in proanthocyanidins from other tannin-rich foods. Howell et al. [75] postulated that this is the reason why cranberry consumption prevents urinary tract infections while other foods containing only B-linked proanthocyanidin do not. There are no data to date on the antiadhesion activity of B-linked proanthocyanidins.

All of the results described above confirm that polyphenols can modulate the composition of the intestinal microbiota and hence indirectly influence their own metabolism and bioavailability.

### Prebiotics

Diets abundant in plant polysaccharides contain resistant starch and oligosaccharides that undergo bacterial fermentation in the intestine. Indigestible by the host, complex polysaccharides are converted by the primary degrader bacteria resulting in the release of a wide range of SCFAs (mainly acetate, propionate and butyrate) and a number of other metabolites (lactate, pyruvate, ethanol, succinate, soluble oligosaccharides, gases) that are the “fuel” for secondary degrader bacteria [76–78]. Changes in the intestinal microbiota observed in individuals that switch to a GFD can be explained by the reduction in polysaccharide intake. High intake of dietary fiber results in a greater SCFA concentration and lower *E. coli* counts in mammalian intestine, while an opposite trend has been shown with low fiber intake [33].

Bacterial fermentation products can be used as nutrients, as well as growth signals for the intestinal epithelium. For example, butyrate (which has prodifferentiation, antiproliferation and antiangiogenic effects on colonocytes [79]) is utilized by those cells as a major energy substrate; acetate is mainly metabolized in human muscle, kidney, heart and brain; and propionate is cleared by the liver [80].

A nondigestible food ingredient that beneficially affects the host by selectively stimulating the growth and/or activity of one or a limited number of bacteria in the colon, and thus improves host health, is called a prebiotic [81]. A more recent definition of a prebiotic is “a selectively fermented ingredient that allows specific changes, both in the composition and/or activity in the gastrointestinal microflora, that confer benefits upon host well-being and health” [82]. In order for a food ingredient to be classified as a prebiotic, it must satisfy four criteria: (1) be neither hydrolyzed nor absorbed in the upper part of the gastrointestinal tract; (2) be a selective substrate for one or a limited number of beneficial bacteria commensal to the colon, which are stimulated to grow and/or are metabolically activated; (3) consequently, be able to alter the colonic flora in favor of a healthier composition; and (4) induce luminal or systemic effects that are beneficial to the host’s health. Among the food ingredients, nondigestible carbohydrates (oligo- and polysaccharides), some peptides and proteins, and certain lipids (both ethers and esters) are candidate prebiotics. Because of their chemical structure, these compounds are not absorbed in the upper part of the gastrointestinal tract nor hydrolyzed by human digestive enzymes. Nondigestible lipids that naturally occur in the diet have not yet been examined in this context, and their metabolism and beneficial effect on host health is still theoretical. In the case of proteins that reach the colon, their anaerobic proteolysis generates a range of metabolites including the branched-chain fatty acids such as isobutyrate, isovalerate and isocaproate, as well as indoles, phenolic compounds, sulfides, ammonium, histamine and oxaloacetate. These putrefactive products are generally considered to be toxic and to cause adverse effects on the colonic epithelium [81, 82], so proteins are not considered viable as prebiotics. Most promising prebiotics are nondigestible oligosaccharides, including fructooligosaccharides, galactooligosaccharides (GOS) and inulin. Trans-galactooligosaccharides are a mixture of oligosaccharides derived from the enzymatic transglycosylation of lactose. The selective growth stimulation of *Bifidobacterium* and *Lactobacillus* by GOS has been shown [83–86] along with a significant decrease in the concentration of *Bacteroides*, *Candida* [83] and *Enterobacteriaceae* [84]. Lactulose, which is one of the best characterized prebiotic components produced by the isomerization of lactose, selectively stimulated growth of *Bifidobacterium*, *Lactobacillus* and *Streptococcus*, whereas numbers of *C. perfringens*, *Bacteroides* and *Enterobacteriaceae* were reduced [82].

Supplementation of fecal specimens with lactulose and blood decreased toxic short-chain (iC4-nC6) fatty acid (isobutyrate, butyrate, isovalerate, valerate, isocaproate and caproate) production and increased acetate and lactate production, resulting in increased fecal acidity. These changes were statistically significant when compared with supplementation with blood alone and correlated with the reduction in the growth of *C. difficile* and *Bacteroides* spp. [87].

Inulins are common plant storage carbohydrates that are nutritionally classified as dietary fibers. Inulin-type fructans are present in a range of different plants including wheat, onion, banana, garlic, leek and *Agave tequilana* [80]. Numerous in vitro and in vivo studies have demonstrated that inulin and oligofructose selectively stimulate the health-promoting groups of human intestinal microbiota, *Bifidobacterium* and *Lactobacilli* [88]. Administration of inulin and oligofructose, either alone or as a synbiotic, has been shown to selectively increase numbers of bifidobacteria in the luminal and mucosa-associated microbiota, typically representing a prebiotic effect [88–90]. Both in vitro and in vivo studies have demonstrated that the colonic fermentation of inulin-type fructans increases the production of butyrate, which is the so-called butyrogenic effect. This can be explained by bacterial cross-feeding; i.e., lactate produced by *Bifidobacterium adolescentis* is converted to butyrate by *Eubacterium hallii* and *Anaerostipes caccae* (both *Firmicutes*). The latter bacterium can also utilize released fructose monomers to produce butyrate [91, 92].

### Polyunsaturated fatty acids

Polyunsaturated fatty acids (PUFAs) include, among others, the ω-3 fatty acids (linolenic, eicosapentaenoic and docosahexaenoic acids) and ω-6 fatty acids (linoleic and arachidonic acids). Some interactions of PUFAs with the gut microbiota or with certain probiotics have been reported. In vitro studies on the effects of free linoleic, γ-linolenic, arachidonic, α-linolenic and docosahexaenoic acids at concentrations of 10–40 μg/ml demonstrated the inhibition of growth and mucus adhesion of the probiotics *L. rhamnosus* GG and *L. casei* Shirota as well as *Lactobacillus delbrueckii* subsp. *bulgaricus* (dairy strain). PUFAs also altered bacterial adhesion sites on Caco-2 cells [93]. In contrast, when linoleic acid and α-linolenic acid were added to medium with various *Bifidobacterium* species, their conversion to conjugated linoleic acid (CLA) and conjugated linolenic acid isomers, respectively, started immediately [94]. There is growing interest in CLA because both beneficial and detrimental effects for human health have been reported [95, 96].

## Diet-derived bioactive metabolites produced by gut microbiota and their potential impact on human health

The intestinal microbiota is equipped with a large set of different enzymes capable of various modifications of food ingredients that enter the colon. It can hydrolyze glycosides, glucuronides, sulfates, amides, esters and lactones through the action of enzymes such as *α*-rhamnosidase, *β*-glucuronidase, *β*-glucosidase, sulfatase and esterases. Other reactions catalyzed by the gut microbial enzymes are aromatic ring cleavage, reductions (reductases, hydrogenases), decarboxylation (decarboxylase), demethylation (demethylase), isomerization (isomerase) and dehydroxylation (dehydroxylase) [97, 98]. This large and diverse enzymatic capacity generates various metabolites that have both beneficial and detrimental effects on the host. In addition to the previously mentioned SCFAs, the gut microflora thus generates many important bioactive metabolites.

### Polyphenols

A vegetarian diet is abundant in polyphenols and antioxidants, which are secondary plant metabolites with many important properties such as anti-inflammatory, antimicrobial, antiradical and anticancer activity [99, 100]. It has also been shown that anthocyanins, important compounds present in various types of berries, can promote urinary tract and dermal health, as well as exhibiting anticancer, antioxidant, neuroprotective, cardioprotective and antidiabetic properties [101, 102]. The bioavailability and impact of polyphenols on the host greatly depend on their transformation by specific components of the gut microbiota via esterase, glucosidase, demethylation, dehydroxylation and decarboxylation activities [97].

Owing to the great diversity of species forming the intestinal microbiota in different individuals, the profile of polyphenol metabolites that are generated and their final effect on the body are highly variable within the human population. In many situations, only a product of bacterial metabolism of a polyphenol can be absorbed and exert a beneficial impact in humans. A lack of particular species within the microbiota may mean that a polyphenol cannot exert its expected effect even though it has been consumed.

**Fig. 1:**
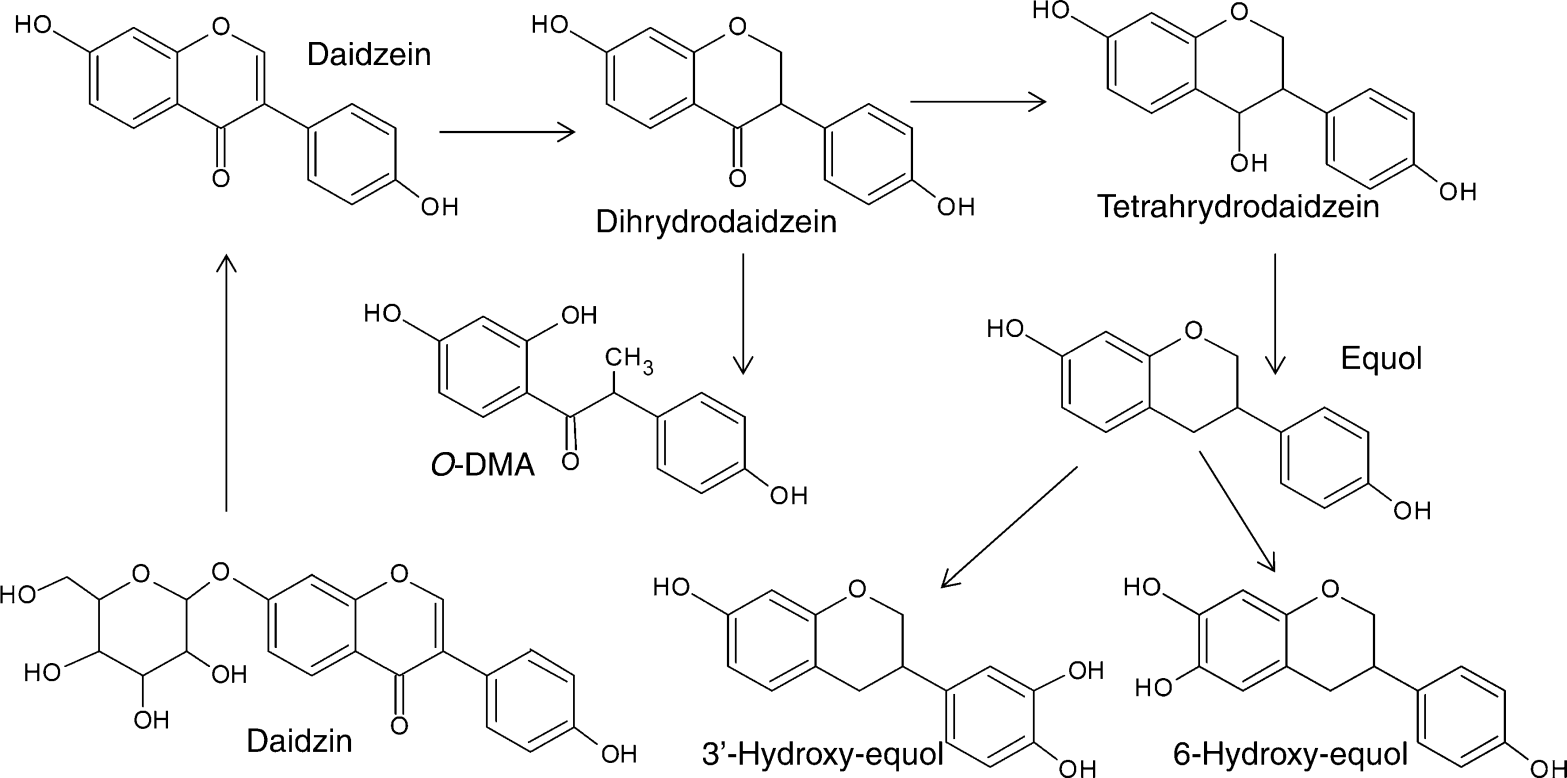
Proposed pathways of bacterial metabolism of daidzin and daidzein (based on [16, 103, 111]) (color figure online)

One of the better-characterized types of polyphenol metabolites are the nonsteroidal estrogens. Some intestinal bacteria, such as *Eggerthella* spp. strain YY7918, *Eggerthella* spp. Julong 732, *Enterococcus faecium*, *Adlercreutzia equolifaciens*, *Slackia equolifaciens*, *Lactobacillus mucosae*, *Bifidobacterium* spp., *Slackia isoflavoniconvertens* and *Bacteroides ovatus* [103–109], are able to metabolize the soya isoflavone daidzein to equol and/or O-desmethylangolensin (O-DMA). The prevalence of equol producers and O-DMA producers is approximately 30–50 and 80–90 %, respectively [108–110], and the rate of equol formation is influenced by dietary habits, food matrix, composition of the intestinal microflora, extent of intestinal bacterial fermentation, intestinal transit time and alterations in the redox level in the large intestine [111]. The metabolic pathways of daidzein degradation by bacteria are presented in Fig. 1.

**Fig. 2:**
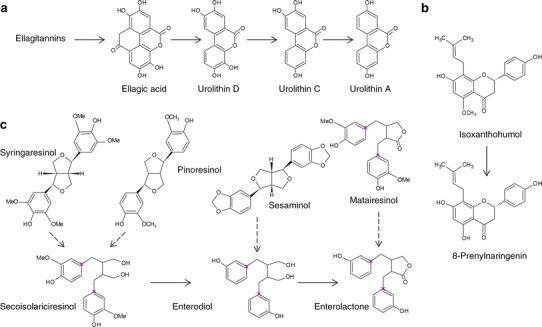
Ligands for estrogen receptors generated by the intestinal microbiota: **a** urolithin A; **b** 8-prenylnaringenin; and **c** enterolactone and enterodiol (based on 16, 97, 120) (color figure online)

Equol exerts many different effects, the most important being endocrine. Its high binding affinity to the estrogen receptor (S-equol preferentially activates ERβ) [108, 109, 112] has been used to alleviate the symptoms of menopause [107]. Antiandrogenic effects and inhibition of osteoclast formation have also been observed [113–115]. In vitro studies have revealed anticancer activities, mainly due to the inhibition of cancer cell migration and invasion, as well as induction of apoptosis in cancer cells [116, 117]. Anti-inflammatory effects are the results of iNOS inhibition and eNOS activation [118]. Other ligands for estrogen receptors are enterolactone, enterodiol, urolithin A and 8-prenylnaringenin. These are polyphenol metabolites generated by various bacteria, e.g., *Bacteroides* spp., *Clostridium* spp., *Eubacterium* *limosum* and *E. lenta* [119–121]. Enterolactone and enterodiol are lignan derivatives of polyphenols from sesame seed or flaxseed, urolithin A is a metabolite of ellagitannins from various berries, while 8-prenylnaringenin is generated from isoxanthohumol present in hops (Fig. 2). All of these can bind to estrogen receptors and inhibit cancer development by the inhibition of cancer proliferation, invasion and angiogenesis [121–123].

**Fig. 3:**
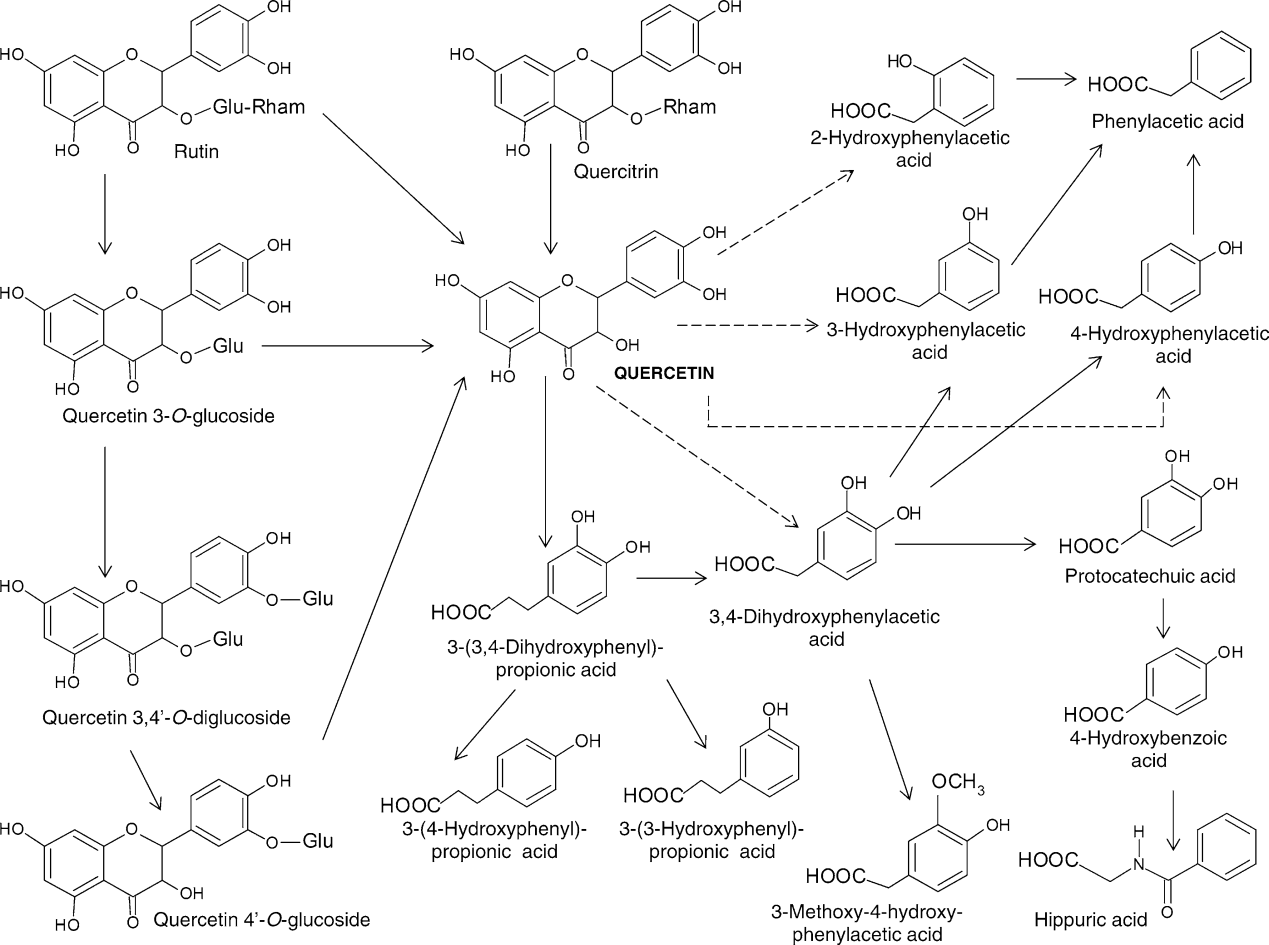
Possible pathways of the transformation of quercetin and its glycosides due to metabolism by intestinal bacteria (based on [16, 138, 156, 157]) (color figure online)

Some polyphenols and products of their metabolism have a negative effect. For example, owing to metabolism by intestinal bacteria, quercetin and rutin can be transformed to 3,4-dihydrophenylacetic acid (DOPAC) (Fig. 3), which is a metabolite of the neurotransmitter dopamine. It has been demonstrated that DOPAC has anticancer, anti-inflammatory, cardioprotective and neuroprotective properties. However, in the presence of the NO radical, DOPAC inhibits mitochondrial respiration in isolated brain mitochondria, leading to mitochondrial dysfunction, which might be an important mechanism involved in the neurodegeneration associated with Parkinson’s disease [124–126].

Procyanidins, oligomers of flavan-3-ols, are present in many fruits, especially in berries, wine, tea and nuts. They can be metabolized by intestinal bacteria to 3,4-dihydroxyphenylvaleric acid, which can be further degraded into phenolic acids such as 3,4-dihydroxyphenylpropionic (dihydrocaffeic acid) and 3,4-benzoic acids. However, recent studies have postulated that α-oxidation of 3,4-dihydroxyphenylvaleric acid can lead to 3,4-dihydroxyphenylacetic acid formation, which means that this compound can be released from both monomeric and dimeric flavonols (procyanidin) [127].

Excessive intake of many polyphenolic compounds seems to exert an adverse effect on the body [128]. First of all, in the presence of O_2_ and transition metals (Cu, Fe), some polyphenols may act as pro-oxidants, leading to damage of DNA, lipids and other biological molecules. This pro-oxidant activity can be applied for cancer therapy and has a beneficial impact on the host cell, but if uncontrolled, the effect will be detrimental. Moreover, flavonoids can change the activity of phase I and II metabolizing enzymes, such as cytochrome P450 (CYP), P-form phenol sulfotransferase, glutathione S-transferase, NAD(P)H:quinone oxidoreductase and UDP-glucuronyl transferase. This inhibitory/stimulatory activity of particular polyphenols is utilized in cancer therapy [128]. However, it has been observed that simultaneous administration of flavonoids and clinically used drugs may cause flavonoid–drug interactions. Such interactions, by modulating the pharmacokinetics of the drugs, can increase their toxicity or diminish their therapeutic effect, depending on the flavonoid structure [128]. Naringenin, a major flavanone present in grapefruit juice, exerts an inhibitory effect on intestinal CYP3A4 within 30 min. This means that the metabolism of certain drugs, such as those belonging to calcium channel antagonist or immunosuppressant groups (e.g., felodipine, nitrendipine, nicardipine, amlodipine, nisoldipine, verapamil, terfenadine, cyclosporine, midazolam, triazolam), would be impaired if they were co-administered with grapefruit juice [129].

The bioactive properties of polyphenol metabolites can be completely different from the activity of the parent compounds. There are approximately 700 anthocyanins isolated from plants, but the most commonly known are based on six anthocyanidins: cyanidin, delphinidin, pelargonidin, peonidin, petunidin and malvidin [130, 131]. The majority of dietary anthocyanins are not absorbed in the upper parts of the gastrointestinal tract, so they reach the colon and are metabolized by intestinal microbiota, resulting in the generation of new compounds, which may be absorbed and thus have an impact on both the microbiota and the host. The bacterial catabolism of anthocyanins involves the cleavage of 3-glycosidic linkages by bacterial β-glucosidase, which causes instability of the anthocyanidin at neutral pH and rapid breakdown of the heterocyclic ring. The major stable products of degradation are corresponding phenolic acids (derived from the B-ring of the anthocyanin skeleton) and phloroglucinol derivatives (from A-ring) [131, 132].

The main phenolic acids found as the colonic metabolites of various anthocyanins are protocatechuic, gallic, syringic, *p*-coumaric and vanillic acids [131]. The other products of anthocyanin degradation by intestinal microbiota are 2,4-dihydroxybenzoic acid and 2,4,6-trihydroxybenzoic acid, which have been detected in in vitro studies [133].

Protocatechuic acid (PCA) is one of the main bacterial metabolites of complex polyphenols such as anthocyanins and procyanidins that are normally found at high concentrations in vegetables and fruit [134]. Available results support the concept that PCA can exert a variety of biological effects by acting on different molecular targets. It has been shown that PCA possesses antioxidant, anti-inflammatory, antihyperglycemic and neuroprotective activities and may thus have a preventative role in various diseases. Moreover, it inhibits in vitro carcinogenesis and exerts proapoptotic and antiproliferative effects in different tissues [135], inhibits monocyte adhesion to tumor necrosis factor-alpha-activated mouse aortic endothelium, exerts an antiatherogenic effect and decreases cholesterol levels [136–139]. The anthocyanin microbial catabolites, such as gallic acid, 3-O-methyl-gallic acid and 2,4,6-trihydroxybenzaldehyde, have been tested for their ability to induce apoptosis and inhibit cell proliferation in a colon cancer model using Caco-2 cells. All of these compounds reduced cell proliferation without being cytotoxic and were more effective than the parent anthocyanins [140]. Other anthocyanin degradation products, 3′-hydroxyphenylacetic acid, 3′,4′-dihydroxyphenylacetic acid and 3′-methoxy-4′-hydroxyphenylacetic acid, inhibited the formation of advanced glycation end-products and were effective in preserving cultivated neuron cells from death due to oxidative stress [141].

Anaerobic bacteria such as *Bacteroides distasonis*, *B. uniformis* and *B. ovatus* can release aglycone from quercetin glycosides owing to their β-glucosidase activity [138]. *Enterobacteria* also decompose quercetin aglycone to produce its ring scission products such as 3,4-dihydroxyphenylacetic acid, m-hydroxyphenylacetic acid and m-homovanillic acid [139]. The aglycone is converted to its conjugated metabolites with or without O-methylation and then transferred to the liver through the portal vein. Some conjugated metabolites are likely to be transferred into the systemic circulation via lymph. Quercetin glucuronides are another kind of quercetin metabolite in humans, and their plasma concentration as well as biological activity depends on the conjugation position. Day et al. [142] demonstrated that the 5-position did not appear to be a site for quercetin conjugation. The K(i) for the inhibition of xanthine oxidase by quercetin glucuronides followed the order 4′- > 3′- > 7- > 3-, with quercetin-4′-glucuronide a particularly potent inhibitor. The glucuronides, with the exception of quercetin-3-glucuronide, were also inhibitors of lipoxygenase. It was demonstrated that quercetin metabolites are likely to exert their physiological function at the target sites where they concentrate. It is highly probable that conjugated metabolites of quercetin circulate as nontoxic forms in the bloodstream, but are later converted to active aglycones to exert their function immediately. This effect was observed by Kawai et al. [143]. In vitro experiments using murine macrophage cell lines showed that quercetin-3-glucuronide (Q3GA) was taken up in significant quantities and deconjugated into the much more active aglycone, a part of which was further converted to the methylated form, in activated macrophages. Q3GA accumulated only in atherosclerotic lesions but not in normal aorta [143].

### Vitamins

The microbiota is involved in the production and absorption of essential vitamins and micronutrients. The increased concentration of SCFAs, which is a final result of bacteria’s metabolism of resistant starch and oligosaccharide, may help in the absorption of minerals such as calcium, by increasing their solubility and increasing the expression of calcium binding proteins [144]. Many studies have shown that resistant carbohydrate can be utilized by the intestinal bacteria for folate biosynthesis. The main producers of folate are *Bifidobacterium bifidum* and *B. longum* subsp. *infantis* [145], *Lactococcus lactis* subsp. *lactis*, *Streptococcus thermophilus* [146], *B. adolescentis*, *B. pseudocatenulatum* [147] and *L. plantarum* [148]. A number of other B-group vitamins are synthesized by members of the gut microbiota, such as thiamine, riboflavin, niacin, pantothenic acid, pyridoxine, biotin and vitamin B12 [145, 149]. There are also some studies that indicate a role of the intestinal microbiota in menaquinone (vitamin K2) production in the human gastrointestinal tract. Major vitamin K producers are species from the *Bacteroides* genus, *E. coli*, as well as some strains of *K. pneumoniae*, *Propionibacterium* and *Eubacterium* species [150]. A negative correlation between a low-fiber diet and the abundance of menaquinone-producing bacteria in the gut has been demonstrated [151].

Among other important bioactive compounds produced by the intestinal microbiota are bacteriocins, factors that inhibit the growth of pathogenic bacteria. Bacteriocin-producing strains of the *Pediococcus* and *Lactococcus* genera have been isolated from human intestine [152]. It has also been shown that human isolates of *Pediococcus acidilactici*-producing pediocin PA-1 and *L. lactis*-producing nisin Z are able to reduce intestinal colonization by vancomycin-resistant enterococci in a mouse model [153]. Moreover, *L. lactis* and *P. acidilactici* administration increased the total number of lactic acid bacteria and anaerobes, while *P. acidilactici* decreased the *Enterobacteriaceae* population.

## Conclusions

For many years, the perception of the function of the large intestine was that it was limited to the reabsorption of water and salt and the removal of unused food debris, completely ignoring the microbiota present. Currently, it is known that a large number of bacteria inhabiting the large intestine form a very complex ecosystem called the ‘intestinal microbiome’. The intestinal microbiome plays an important, possibly crucial, role in the metabolism of chemical compounds found in foods. It is estimated that its metabolic capacity is approximately 100-fold greater than the capacity of the liver, because of the great diversity of bacterial species forming the population, and hence the large number of genes that they contain [4]. Moreover, the enormous total number of microorganisms that inhabit the large intestine has considerable influence. There are approximately 10^12^ bacteria per gram of colonic content, which is the highest accumulation of microorganisms that has ever been noted in any environment [13].

Owing to the multitude of direct and indirect interactions with the host organism, the intestinal microbiome is closely linked to the health of the host [15]. The microbiota plays a protective role by occupying intestinal surfaces and creating a milieu that prevents the invasion of pathogens (e.g., by production of bacteriocins and other antimicrobial compounds). Moreover, the metabolism of those bacteria enables the breakdown of indigestible compounds such as resistant starch and plant polysaccharides during anaerobic fermentation, which results in SCFA production. Other examples of bacterial metabolism are vitamin and amino acid synthesis [14]. These metabolites can then serve as growth signals and nourishment for the intestinal epithelium of the host, as well as for other microorganisms. Furthermore, the etiology of many diseases is largely correlated with the intestinal microbiome or certain microbial members. A balance between the host immune system and the commensal gut microbiota is crucial for maintaining health. If this balance is disturbed (dysbiosis), the host–microbe relationship can progress toward a disease state [14, 25]. Recent studies suggest that specific members of the gut microbiota play a functional role in inflammatory bowel disease (including Crohn’s disease), colorectal cancers, obesity and allergies [4, 14, 15, 22, 28, 97, 154, 155].

Over the course of evolution, the human organism has developed tools indispensable for the utilization of sugars, lipids and proteins. Despite this, many of these compounds enter further segments of the gastrointestinal tract undigested (intact), where they interact with the intestinal microbiota. Bacteria use these compounds as a source of energy and nutrients via their own enzymes, some of which cannot be produced by human tissues. It should be remembered that many of these substances have antibacterial properties. This enables the elimination of pathogens, but may have also a detrimental effect due to elimination of beneficial microflora. Typically, xenobiotics undergo biotransformation processes via the intestinal bacteria, which are necessary for their detoxification. The potential of the intestinal microbiome for detoxification or bioactivation of xenobiotics is much higher than that of the liver and includes other types of reactions not found in the liver. Hepatic metabolism is based on the reactions of oxidation and conjugation, mainly resulting in the generation of hydrophilic high molecular weight metabolites. Bacterial metabolism occurs under anaerobic conditions and is based mainly on reduction and hydrolysis reactions, resulting in nonpolar low molecular weight products. Therefore, bacterial metabolites are different from those that can be generated by human enzymes, and their impact on both host health and intestinal microbiota thus differs.

Bacterial metabolism usually reduces the activity of dietary compounds such as polyphenols, but sometimes a specific product of bacterial transformation exhibits enhanced properties. The products of bacterial metabolism may exhibit enhanced or more beneficial effects, or they may be degraded to inactive or toxic compounds. Studies on the metabolism of polyphenols by the intestinal microbiota are therefore crucial for understanding the role of these compounds and their effects on our health.
